# Use of planar covariation in lower limb kinematics to characterize adaptations of running after cycling in elite triathletes

**DOI:** 10.3389/fspor.2022.1047369

**Published:** 2023-01-10

**Authors:** Joel A. Walsh, Alexander Stamenkovic, James P. Dawber, Paul J. Stapley

**Affiliations:** ^1^ Neural Control of Movement Laboratory, School of Medical, Indigenous and Health Sciences, Faculty of Science, Medicine and Health, University of Wollongong, Wollongong, NSW, Australia; ^2^ Motor Control Laboratory, Department of Physical Therapy, Virginia Commonwealth University, Richmond, VA, United States; ^3^ Southampton Statistical Sciences Research Institute, Social Statistics & Demography, University of Southampton, Southampton, United Kingdom

**Keywords:** intersegmental coordination, triathlon, kinematics, running, cycling

## Abstract

**Purpose:**

To characterize alterations of lower limb intersegmental coordination during the acute phase of running after cycling among highly trained triathletes using an analysis of planar covariation.

**Methods:**

Nine highly trained triathletes completed a control run (CR) and a run after transitioning from cycling exercise (transition run, or TR condition) on a motorized treadmill at a self-selected pace. Sagittal plane kinematics were recorded using a 3D Vicon motion capture system. Intersegmental coordination of the thigh, shank and foot segments of the right lower limb and run loop planarity were calculated during running before cycling and at four different times after the end of cycling.

**Results:**

PCA showed a significant within-subject phase shift of the run loop planarity (*F* = 6.66, *P* = 0.01). *Post hoc* analysis showed significance median differences increase for *u*_3t_ parameter between CR_SS_ vs. TR_30_ (*P* = 0.01), TR_t1/2_ (*P* = 0.01) and TR_MRT_ (*P* = 0.01). No difference for *u*_3t_ parameter existed between CR_SS_ vs. TR_SS_.

**Conclusion:**

Prior variable-cadence, moderate intensity cycling has a significant effect on run loop planarity and therefore intersegmental coordination during the acute transition phase among highly trained triathletes. However, alterations to lower limb coordination are corrected by the 3rd minute after the beginning of the post cycle run. We suggest that planar covariation can be used as a more sensitive measure of cycling-induced variations in running to characterize adaptation in elite and importantly, developing athletes.

## Introduction

In triathlon and duathlon, the transition between cycling and running involves a rapid change between well learned modes of locomotion: cycling and running. With ever-improving run times in triathlon and an evolution of race format to multiple combinations of swim/bike/run, this acute post cycle adaptation of running immediately after the second transition period (T2) in triathlon from the onset to steady state running is becoming more and more crucial to overall race performance.

In amateur triathletes, studies have reported that prior cycling significantly affects muscle recruitment patterns and lower limb joint kinematics during subsequent running (1–5). Specifically, Rendos et al. (4) reported increases in anterior pelvic tilt and hip flexion during running after 30 min of moderate intensity cycling compared to running without having previously cycled (control running, CR). However, among elite triathletes, lower limb joint kinematics and muscle recruitment patterns have been reported to be largely unaffected during the period immediately post T2, when compared to CR patterns (6, 7). Interpretation of joint angle changes have been limited to analysis of lower limb joint displacements over time which may not provide sufficient detail of impaired intersegmental coordination. In contrast, temporal changes of the angles of lower limb segments (i.e., thigh, shank and foot) with respect to an external reference such as the gravitational vertical relate to the economy of movement (8, 9). These so-called “elevation angles” of lower limb segments can be used to characterize inter-segmental coordination during walking and running (8). Therefore, an interpretation of the relationships between joint angles reflecting movement economy at specific physiological timepoints in the acute phase of running, post cycling may inform us as to how altered intersegmental coordination is adapted to through training in elite triathletes.

During locomotion (i.e., walking and running) lower limb segments (i.e., thigh, shank and foot) are strongly coupled (9), so that the three-dimensional timing of intersegmental coordination runs along a specific plane of angular covariation (10). This is known as planar covariation (10, 11). Numerous authors have suggested that the planar covariation of the thigh, shank and foot segments rationalize human locomotion control (9, 12) and provides intelligence regarding the nervous system's control of muscle activation strategies (10, 13, 14).

This approach of analyzing intersegmental coordination could prove useful in analyzing movement efficiency when transitioning between two different movement tasks, especially when overlapping of generalized movement patterns could potentially interfere with neural control strategies governing these movement tasks (15). The goal of this study was therefore to investigate intersegmental coordination of lower limb kinematics using a planar covariation analysis of running during the acute phase post T2, compared to CR, among highly trained elite triathletes. We applied a technique of characterizing the planar covariation of elevation angles of the lower limb at specific times based on VO2 kinetics (7) from onset to steady state running immediately post cycling. The chosen times enable us to match up kinematic measures of economy of movement to indicative physiological timepoints after the transition from cycling to running. Our results show that this technique can identify specific changes in running in elite triathletes after cycling that may not be identified using standard analyses of angular displacements.

## Methods

### Participants

Nine triathletes (males = 5, females = 4: age: 24.3 ± 7.6 years; mass: 67 ± 9.7 kg; height 175.6 ± 8.2 cm; BMI 21.6 ± 1.9 kg/m^2^) who had experienced National level and/or World Triathlon level competition (3.3 ± 1.3 years) during the year of testing participated voluntarily in this study. Each participant was considered “highly trained” according to the definition of Chapman et al. (1, 15). Participants maintained their current training regime leading up to the study but refrained from completing any training or vigorous exercise on the day of testing. Each participant provided their written informed consent prior to partaking in the study. All experimental procedures and methods were approved by the university's human research ethics committee (HE12/331) and conducted in accordance with the Code of Ethics of the World Medical Association outlined in the Declaration of Helsinki.

### Experimental procedures

As in previous studies (7, 16) we adopted an experimental protocol developed to minimize the accumulation of fatigue using moderate-intensity cycling and running in order to analyze neuromuscular changes when running after cycling (15). All participants completed a standardized 5-minute warm-up run during which they self-selected a running speed. Mean running speed for all participants was 12.9 ± 1.8 km/h. All runs were undertaken on a motorized treadmill (Landice, Randolph, NJ).

Following 10-min of recovery participants completed a 10-min CR at their self-selected speed. After 60-min of seated recovery, subjects completed 20-min of continuous, variable-cadence (55–100 rpm) cycling with their personal racing bikes (road bike) mounted on a magnetic cycle ergometer (Tacx Satori Trainer, Tacx, Netherlands). Cycling cadences were controlled using a Garmin® 500 ANT+ bike computer and were based on those used by Chapman et al. (17). Each subject transitioned from cycling to running during a phase of <60 s and completed a subsequent 10-min transition run (TR), again at their self-selected speed.

The CR and TR running tests were conducted on a motorized treadmill (Landice, Randolph, USA) at a 0% grade, consistent with previous studies (1, 6). Treadmill speed was increased at increments of 0.5 km/h to the required self-selected speed that was reached within the first 20 s of commencing running. Ratings of perceived exertion (RPE) were used to control cycling exercise intensity using the Borg 15-point (6–20) RPE scale, known to be a validated and reliable method of regulating intensity during cycling and running (18, 19). Participants were instructed to maintain a constant cycling exercise intensity at a RPE of 14. RPE was recorded (participant was asked to verbalise it when shown a scale) every 1 min during both CR and TR runs and participants were requested to also maintain a RPE of 14 while they ran at the same speed as in the CR.

Three-dimensional, sagittal plane kinematic data of the lower limbs were collected during the CR and TR trials using a five camera Vicon Bonita (ViconPeak, Oxford Metrics Ltd., Oxford, UK) motion analysis system (240 Hz). Sixteen 15-mm reflective markers were affixed to the skin using double-sided tape and Fixomull® (BSN Medical, Germany) over anatomical locations on the pelvis (left and right ASIS and PSIS), lower limb (lateral thigh, lateral epicondyle, lateral tibia, lateral malleolus) and foot (the posterior calcaneus and 2nd metatarsal head). Markers positioned over the posterior calcaneus and second metatarsal head were fixed to each participant's shoes. A Vicon Plug-in-Gait® labelling template and functional skeleton were overlayed onto the range of motion (ROM) calibration trials. All kinematic data underwent Vicon pipeline processing where marker trajectories were pattern gap-filled before being Woltring filtered and exported (.c3d file) for off-line analysis using MATLAB (Version 2013b, The MathWorks Inc. Natick, MA, USA).

### Physiological timepoints for the determination of kinematic measurements

Three-dimensional kinematic data of the right lower limb was collected at the same time as pulmonary gas exchange and ventilation (breath-by-breath VO_2_) using a metabolic gas analysis cart (Parvo TrueMax 2400, Parvomedics, Sandy, UT, USA). Kinematics were quantified from 20 running run cycles (10 cycles before and after the respective specific physiological event) at 5 different timepoints. The timepoints corresponded to previously published VO_2_ measures (explained in Walsh et al., 2015, 2017) and were: (1) the start of the 10th minute of the control run (CR steady state, or CR_SS_), (2) 30 s after reaching the pre-cycle CR self-selected speed (TR_30_), (3) theoretical “half-time” to steady state VO_2_ calculated using a remodelled logarithm (20), TR_t1/2_, (4) mean response time (TR_MRT_) defined as the time required to reach about 63% of steady state (21), and (5) at the beginning of the 3rd minute when running after cycling [steady state or TR_SS_, (4)]. Run cycles were defined using visual inspection of the kinematics of running as the period between heel strike to ipsilateral heel strike (0%–100%), that were determined by identifying the point of heel strike to toe-off of the right foot (22).

### Planar covariation calculations from 3D kinematics of the lower limb

**Figure 1:**
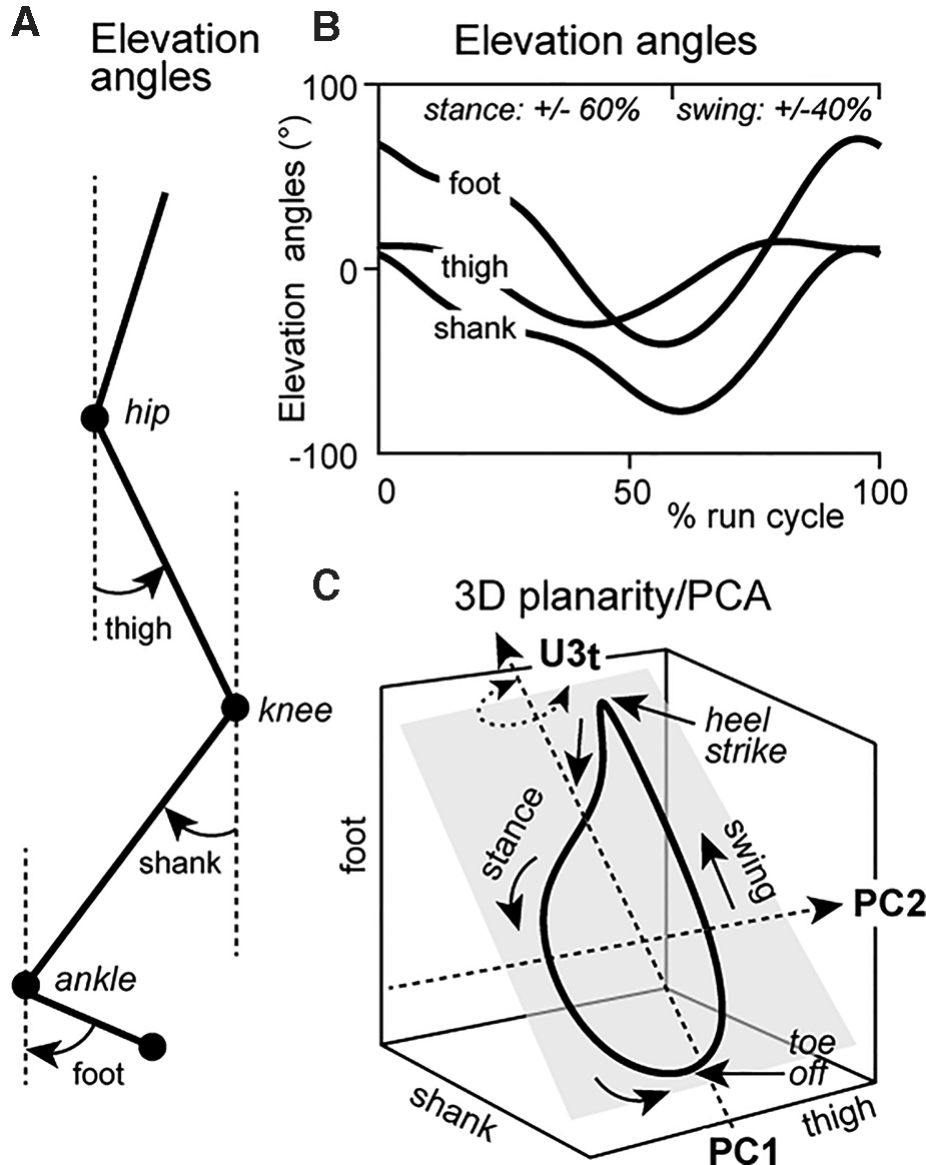
(**A**) Representation of the joint angles of the thigh, shank and foot with reference to the line of gravity—elevation angles; (**B**) Sagittal plane elevation angles of joint waveforms through a run cycle (stance = ∼0%–60% and swing = ∼60%–100% of run cycle). (**C**) Elevation angles of the thigh, shank and foot plotted as thigh × shank × foot, representing 3D planar covariation. Evolution of the run cycle progresses in a counterclockwise direction. Heel strike and toe-off correspond to the top and bottom of the run loop, respectively. Changes to the *u*_3t_ parameter are represented by rotation of the planar orientation of the run loop (grey square) about the long axis. Rotation of the run loop remains perpendicular to the thigh axis.

Angles of the thigh, shank and foot relative to the vertical were calculated (Figures 1A,B, “Elevation angles”). Elevation angles were computed using a custom written MATLAB script to analyze planar covariation parameters during the CR and TR trials, in accordance with the methodology outlined by Borghese et al. (23, 24) and Bianchi et al. (22). Intersegmental coordination parameters were calculated by principal component analysis (PCA) on elevation angles for each gait cycle separately to obtain three principal components with corresponding variabilities PV_1_, PV_2_, and PV_3_ and eigenvectors *u*_1_, *u*_2_, and *u*_3_, as originally described by Borghese et al. (23, 24) and Bianchi et al. (22). Temporal progression of the run loop occurred in a counter-clockwise direction with heel strike and toe-off phases being analogous to the top and bottom of the run loop, respectively (Figure 1C). Percentage of variation for the first and second principal components (PV_1_, PV_2_) determined the best-fitting plane of angular covariation (10). Plane orientation was defined and plotted by the third component (PV_3_) for CR and TR conditions.

We specifically analyzed the *u*_3t_ parameter for PV_3_ of the thigh segment during the CR and TR conditions. *u*_3t_ was defined as the direction cosine corresponding to the 3rd eigenvector (“3”) in the thigh (“*t*”) angular elevation axis. This parameter characterizes the orientation of the normal plane of angular covariation about the long axis of a gait cycle (8, 23) and perpendicular to the thigh axis (10) (Figure 1C). For example, a *u*_3t_ value of 0 would indicate no rotational change of the plane of angular covariation of the thigh (10). This is important as the closer to zero a value of *u*_3t_ is, the less important it becomes in explaining the high planar covariation between the shank and foot (i.e., coordination is simplified), which in turn indicates a more efficient movement economy as the thigh would not exert a strong influence on the whole lower limb coordination pattern. At equitable speeds, a *u*_3t_ value closer to zero suggests the lesser the plane is rotated and the lower the energy expenditure, indicating a more efficient movement (8).

### Statistical analysis

To compare the effect of prior variable-cadence cycling on running, median values for each variable (PV_1_, PV_2_, PV_3_) per subject were selected from the run cycle range (i.e., CR_SS_ = 86 ± 3 and TR_30-SS_ = 25 run cycles) and used for analysis in order to reduce measurement error within subject and condition. *u*_3t_ values were also adjusted to CR_ss_ values so that changes were correctly identified to a control run measure for each participant. Difference in stride rates, between CR and TR, were determined using a paired *t*-test. Repeated measures ANOVA was used to test for differences in mean data between conditions, calculated from subject median values for each condition, taking into account repeated measures within subjects. *Post hoc* analysis was carried out using Tukey's HSD. Diagnostic examination was conducted using Shapiro-Wilk analysis to test the normality assumptions of the data model. All statistical analysis was carried out using *R* Studio software (Version 3.2.4, *R* Foundation for Statistical Computing, Vienna, Austria) with significance set at 0.05. Descriptive data are presented as mean ± standard deviation and statistical data is presented with effect size (*ES*) and significance level.

## Results

**Figure 2:**
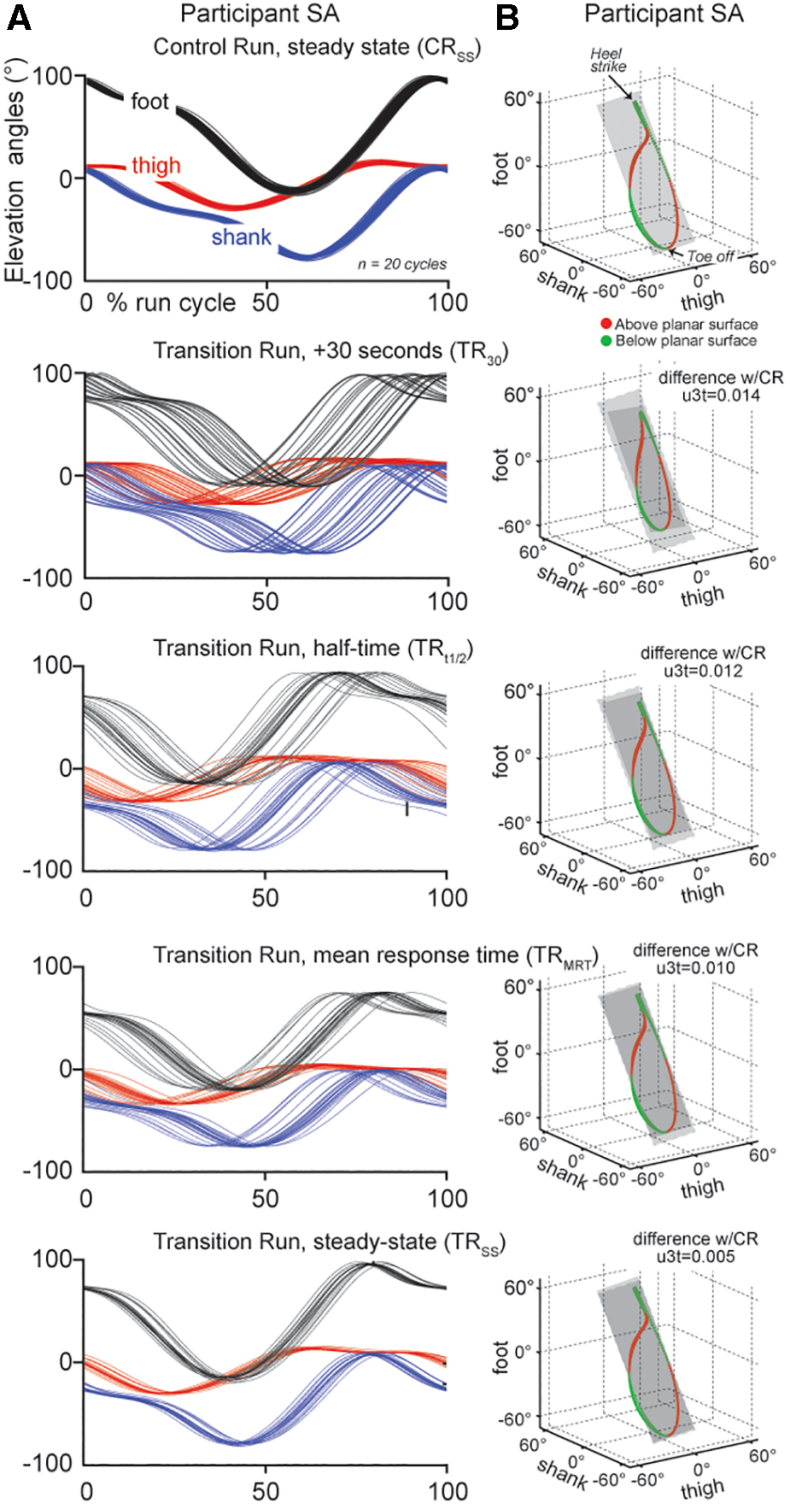
(**A**) Elevation angles for the thigh (red), shank (blue) and foot (black), of a single participant (SA), at all sample times. (**B**) Run loops for a single subject that are representative of the group; red denotes the portion of the run loop that is above the plane of angular covariation (grey panel), with green denoting that the portion of the run loop sits below the plane. The grey panel represents the orientation the plane of angular covariation. Each is shown relative to the control run (steady state) planar covariation and differences in U3t values are shown relative to the CR value for this particular participant (insert to the right of each run loop).

Stride rates did not vary between the CR and TR (CR = 41.1 ± 1.5 and TR 40.9 ± 1.3 strides/min^−1^, *P* = 0.21: *d* = 0.15). Figure 2A shows the typical behavior of elevation angles of the thigh, shank and foot and run loops at the CR_SS_, TR_30_, TR_t1/2_, TR_MRT_, and TR_SS_ time periods for a single participant. The mean percentage of variation for PV_1_, PV_2_ and PV_3_ showed no significant change (*F* = 1.18, *P* = 0.34; *F* = 1.38, *P* = 0.26 and *F* = 1.09, *P* = 0.38, respectively) between the CR and TR conditions indicating that the segments of the lower limb covaried strictly along a plane regardless of prior cycling.

Despite a significant negative correlation (*r *= −0.96, *P* = 0.01), indicating strict covariation of the PC_1_ and PC_2_ principal components, changes in the elevation angles of the shank and foot were observed across the run cycle at TR_30_, TR_t1/2_, TR_MRT_ (Figure 2A). Analysis of the run loop planarity (Figure 2B) between the CR and TR conditions showed a significant within-subject effect for condition on the *u*_3t_ parameter for PV_3_ (*F* = 6.66, *P* = 0.01) due to the progressive phase shift of the shank and foot elevation angles (Figure 2A).

**Figure 3:**
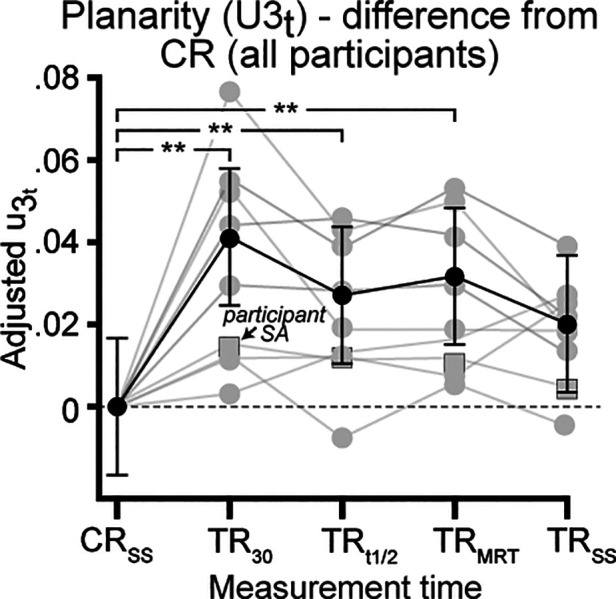
Differences in planarity (*u*_3t_) adjusted to control run (CR) values at all physiological measurement times (see Methods for details). Individual (grey) and group mean (black) adjusted median difference values for parameter of the thigh segment for all participants. Error bars for group mean represent 95% confidence interval. **Significantly different at *P* < 0.01.

*Post hoc* analysis revealed significant median differences increase for *u*_3t_ parameter, when mean adjusted to zero (Figure 3) between CR_SS_ vs. TR_30_ (*ES* = 0.026, *Z* = 4.87, *P* = 0.01), CR_SS_ vs. TR_t1/2_ (*ES* = 0.017, *Z* = 3.19, *P* = 0.01) and CR_SS_ vs. TR_MRT_ (*ES* = 0.020, *Z* = 3.74, *P* = 0.01). The increase in *u*_3t_ parameter represented a rightward rotation of the plane of angular covariation (*u*_3t_ value >0), suggesting that the thigh segment was affected during the acute TR phase. No difference for *u*_3t_ parameter existed between CR_SS_ vs. TR_SS_, as median *u*_3t_ values approach mean adjusted zero.

## Discussion

The current study used PCA to calculate planar covariation of elevation angles of the thigh, shank and foot to investigate temporal changes of intersegmental coordination during the acute TR phase, among elite triathletes. In some participants, prior cycling induced a significant rotational change to the plane of angular covariation of the run loop, as represented by the increased mean adjusted *u*_3t_ value of the thigh segment, during the acute TR phase. Planarity of the run loops at TR_30_, TR_t1/2_ and TR_MRT_ rotated relative to CR_SS_, indicating altered coordination of the thigh segment. These outcomes support the idea that prior cycling can negatively alter intersegmental coordination of the lower limb, even among some highly trained triathletes, during acute TR phases associated with physiologically relevant markers of energy expenditure. However, the *u*_3t_ parameter at CR_SS_ and TR_SS_ showed no significant change, representing a correction of altered intersegmental coordination. Overall, the analysis showed that lower limb coordination of highly trained triathletes can be altered during the acute TR phase however, with athletes being able to rapidly correct altered coordination when transitioning between cycling and running. This strategy would limit energy expenditure in order to maximize endurance performance (9).

At TR_30_, TR_t1/2_ and TR_MRT_ the relative rightward rotation of the run loop represents a negative effect on TR kinematics. This is consistent with previous literature stating that among moderately trained triathletes high intensity (2, 3) and prolonged (∼3 h) (5) cycling has a significantly negative effect on lower limb joint kinematics during subsequent running. Similarly, Rendos et al. (4) showed that 30-min of moderate intensity cycling (RPE 12–14) significantly increased anterior pelvic tilt, hip flexion and spine extension at specific time points (minutes 2, 6, 10 and 14) during the TR, compared to a CR at the same intensity. Moreover, Gottschall and Palmer (25) suggested that preservation of neural control mechanisms coordinating cycling, particularly fast (109 ± 1.91 rpm) cadence cycling, influences running performance and kinematics.

Our results are in contrast to previous studies indicating that neuromuscular control and lower limb joint kinematics, during the 1st–6th minute of the TR, are preserved among elite and highly trained triathletes (6, 22). This may be due to the TR speeds used in this study (i.e., 12.9 ± 1.8 km/h vs. 18 km/h for males and 16 km/h for females, respectively) as locomotor speed influences intersegmental coordination (8, 10). The differences at TR_30_, TR_t1/2_ and TR_MRT_ for the current study and those reported by Chapman et al. (6, 22) and Bonacci et al. (6, 22). could also be related to the sensitivity of the kinematic measurements. Therefore, using PCA to determine intersegmental coordination provides an additional level of sensitivity regarding central nervous system control during locomotion (23, 24, 26) and reflects neural control adaptations endured when transitioning between movement patterns (11). As such, PCA offers a feasible means of detecting small alterations to lower limb coordination during the acute TR phase and could be used to improve marginal performance gains among highly trained triathletes as well as the ability of developing athletes to transition effectively.

Methodological limitations should be considered when interpreting the findings of the current study. First, this study purposely used a moderate-intensity (RPE ∼14) testing protocol in order to minimize any influence of fatigue on the TR (15). As a consequence, caution should be taken if equating these findings to race situations where athletes compete at higher intensities, as planar covariation of elevation angles changes with increasing movement speeds (10). Considering that intersegmental coordination is affected by locomotor speed, it could be hypothesized that alterations to lower limb coordination would be amplified at higher cycle-run intensities. Future studies should use cycle-run protocols that reflect racing demands in order to provide a clearer indication of alterations to lower limb coordination during running, within the acute TR phase. Secondly, the use of a constant, self-selected running speed for both the CR and TR does not account for fluctuations in running speeds reported during triathlon (6).

## Conclusion

Elite triathletes show cycle-induced changes in inter-segmental coordination but are able to correct them by the 3rd minute (i.e., TR_SS_) of the acute TR phase. Neural control of segment rotation is crucial to governing the body's center of mass during locomotion and therefore, conserving energy (8, 27). As such, highly trained subjects appear better able to replicate coupling between limb segments during locomotion in order to save energy (8). Correcting altered lower limb coordination is likely achieved by modulation of neural networks within the spinal cord that govern movement patterns (9, 11), possibly resulting from training and/or experience associated with being a highly trained triathlete. The measures of intersegmental coordination presented in this study improve the ability to identify altered coordination over traditional approaches. Our findings have practical applications for sports clinicians, scientists and coaches by providing an analysis tool for evaluating the effectiveness of training programs and to able to prescribe sessions that specifically focus on improving the ability of multi-sport athletes (i.e., triathletes and duathletes) to transition efficiently from cycling to running and to track the progress of developing athletes.

## Data Availability

The raw data supporting the conclusions of this article will be made available by the authors, without undue reservation.
